# Gambling harms, stigmatisation and discrimination: A qualitative naturalistic forum analysis

**DOI:** 10.1371/journal.pone.0315377

**Published:** 2024-12-10

**Authors:** Katy Penfold, Laura Louise Nicklin, Darren Chadwick, Joanne Lloyd

**Affiliations:** 1 School of Psychology, University of Wolverhampton, Wolverhampton, West Midlands, United Kingdom; 2 School of Education, University of Wolverhampton, Walsall, West Midlands, United Kingdom; 3 School of Psychology, Liverpool John Moores University, Liverpool, Merseyside, United Kingdom; Universidad de Granada, SPAIN

## Abstract

People who experience gambling harms commonly experience stigmatisation, which is detrimental to psychological wellbeing, and a significant barrier to help-seeking. While there have been efforts to challenge stigmatisation, there is little empirical evidence available to inform such initiatives. To address this gap in knowledge, we conducted a thematic analysis of naturalistic data in the form of posts made on online support forums by people with experience of gambling-related harm, in order to understand how they are stigmatised, and to identify barriers to help-seeking. Five main themes were identified: (a) beliefs about the nature and origin of gambling addiction, which related to participants’ beliefs about causes of gambling harm and cognitions about the nature of addiction; (b) self-stigma, which encompassed the frequent and substantial incidences of self-stigma; (c) anticipated stigma, which described the stigma and discrimination people expected to face because of their gambling harm; (d) stigmatising other people who experience gambling harm, which describes the ways in which some people who experienced gambling harms stigmatised other people who experienced gambling harms; and (e) experienced stigma and discrimination, which encompassed the experienced stigmatisation people encountered. Experiences discussed/described within the forums were developed into a timeline of gambling harms which was cyclical in nature and involved six stages: onset, concealment of problems, crisis point, disclosure of problems, recurrence of harms (sometimes termed ‘relapse’) and recovery. The study highlights the impact of societal stigma on individuals’ self-perception and interactions, particularly emphasising the challenges experienced during relapse periods, which heighten stigma and distress. The study also identifies potential avenues for stigma reduction, including targeted campaigns addressing societal, anticipated, and self-stigma.

## Introduction

Stigma has been defined as an attribute that is significantly discrediting or discreditable, especially in the context of relationships [1]. Later, Link and Phelan [2] expanded the definition to include discrimination, highlighting that stigma is not a static attribute but rather one imposed by others. Stigma can be related to a physical characteristic (e.g. deformation), group identity (e.g. race or religion) or personal traits perceived to be rooted in character flaws (e.g. drug addiction, mental illness, ‘problem gambling’; [1].

Stigma relating to various health conditions (e.g. HIV, cancer, and obesity), identities (e.g. gender, sexuality, race), behaviours (e.g. sexual practices, drug use) and occupations (e.g. sex work) has been well-studied over the last few decades, and the negative impact of stigma on physical and psychological wellbeing is well-documented; there is however a dearth of research relating to the stigma associated with gambling harm, despite it being a common experience of those experiencing gambling harms [3].

Stigmatisation is detrimental to wellbeing and associated with a range of psychological difficulties including stress, anxiety, and depression, [4–6]. Moreover, the social isolation resulting from stigmatisation can lead to reduced feelings of belonging and self-worth [7]). Stigma not only affects individuals on a personal level but can also act as a barrier to important resources, such as healthcare and employment opportunities, perpetuating social and economic disparities [8, 9]. Furthermore, the fear of being stigmatised may deter individuals from seeking help and disclosure, hindering effective mental health interventions [10].

Stigma occurs in different ways, including public stigma, perceived stigma, self-stigma (or internalised stigma), anticipated stigma, and experienced stigma and discrimination. Public stigma relates to how society reacts to stigmatised individuals based on negative attitudes towards a stigmatised population or group (e.g. [2, 11–13]). Perceived stigma involves being conscious of public stigma, or a belief that others may hold negative stereotypical attitudes and opinions regarding a specific condition or behaviour [13, 14]. Self-stigma (or internalised stigma) refers to the belief that negative stereotypes about a specific condition or behaviour are true and are applied to the self [15]. Self-stigma is rooted in public stigma, and affects people’s subjective identity, their feelings of self-worth and self-esteem [13]. Anticipated stigma relates to a fear of being judged or of negative reactions in the future [16]. Experienced stigma refers to being stigmatised by others (e.g. [14]), which often results in discrimination whereby stigmatising views are acted on to the detriment of the stigmatised person, for example by excluding or distancing from people who experience gambling harms [17].

There is also a causal link between stereotyping and the perpetuation of stigma. Stereotypes are generalised beliefs about specific groups of people (and an expectation that people might have about every member of that group), which are fixed, oversimplified, often exaggerated and frequently linked to negative traits of behaviours. These beliefs are deeply rooted in societal attitudes. For example, the mainstream media regularly portrays people with mental illness as dangerous, unpredictable or incompetent [18]. These portrayals contribute to stigmatising those with mental health conditions, reinforcing negative attitudes and discriminatory actions against them. Corrigan and Watson [12] demonstrated that exposure to such stereotypes can result in internalised stigma which has a detrimental impact on self-esteem and acts as a barrier to help-seeking. Furthermore, stereotypes influence how others perceive and interact with individuals who experience mental health issues, ultimately leading to social exclusion and marginalisation [19].

The different types of stigma are interconnected and can reinforce each other. For instance, experiencing external stigma may contribute to the internalisation of negative beliefs about oneself. Additionally, the intensity and impact of stigma can vary depending on the specific condition or identity in question, the way that condition is framed, or the extent to which the stigmatised individual is blamed for the characteristic (e.g. [20]). Thus, there is a complex interplay between beliefs about the origin/cause of a condition, perceptions of ‘blame’, and processes of stigmatisation.

In the context of gambling, stigmatisation refers to the social disapproval, discrimination, or negative perceptions directed towards individuals or groups who experience gambling-related harms. Suurvali and colleagues [21] found that individuals experiencing gambling problems reported feelings of shame and embarrassment, leading to social withdrawal and avoidance of seeking help due to fear of judgment and stigma. A study by Hing and colleagues [13] highlighted that gambling-related stigma can act as a significant barrier to help-seeking behaviour, with affected individuals reluctant to disclose their gambling issues or seek professional assistance due to concerns about stigma and negative social consequences. Furthermore, in their analysis of the impact of gambling stigma on treatment-seeking behaviour, Pickering and colleagues [22] noted that individuals experiencing gambling harms often face negative labelling and stereotyping, which can further perpetuate feelings of shame and deter them from seeking support.

As well as the impact on psychological wellbeing, stigma - and the shame associated with it—has been identified as a major barrier to help-seeking for gambling harm [23–27]. Indeed, it is estimated that less than 10% of people who experience harms related to gambling seek formal help [28], which poses challenges for effective intervention and support.

While there have been efforts to challenge stigmatisation (e.g. GambleAware’s 2023 campaign; [29], there is relatively little empirical evidence available to inform such initiatives, for instance around who is most heavily impacted by stigma, and where that stigma originates, or by whom it is most likely to be perpetuated. Furthermore, although it is known that ‘multiply marginalised’ populations are at risk of ‘intersectional stigma’ (i.e. stigma amplified by belonging to multiple stigmatised/ marginalised groups; [30], how this operates in relation to gambling harms is heavily understudied. An additional shortcoming of the current literature is that the little research that has been done has often focussed on individuals receiving formal help, and thus is likely to miss the experiences of the majority of people experiencing harm.

Researching stigma can present many challenges. For example, stigmatised topics are often accompanied by societal taboos, making it challenging for individuals to openly discuss or disclose their experiences, feelings, or behaviours related to stigma [2]; meaning that people may underreport or conceal their experiences with stigma due to fear of judgment, discrimination, or reprisal, [31]. Furthermore, people who hold stigmatising views may hide them for several reasons, such as because they are aware their views are socially undesirable, and thus fear judgement or repercussions if they express them [32, 33]. They may also hold them out of a concern for others as they recognise their views may be offensive or hurtful [34]. Indeed, despite stigmatisation being commonly experienced by people who experience gambling harms [3], a systematic review of the perception of ‘persons with gambling problems’ by Wöhr and Wuketich [35] identified very few incidences of open discrimination. They did however reveal high levels of self-stigma, which was associated with the existence of societal stigma.

One way to circumvent these challenges and uncover the experiences of those not captured by formal help-seeking, is to explore the experiences of those using public online support services such as peer support forums. These forums—such as those hosted by gambling support service providers—offer a level of anonymity which make them particularly appealing to those hesitant to disclose their experiences in more traditional settings [13], and have been identified as effective in helping people to better understand and cope with their problems, as well as alleviating feelings of isolation and building a sense of community among individuals who experience gambling harm [36, 37]. The 24/7 availability of these services is particularly valuable during crises [38]. Moreover, online resources contribute significantly to education and awareness, playing a pivotal role in reducing stigma and fostering a sense of community [36].

From a research perspective, forums are rich repositories of insights into people’s experiences, and their thoughts and feelings about these experiences. They afford valuable naturalistic data [39], and their anonymity reduces likelihood of ‘socially desirable responding’ which can be particularly problematic in research into sensitive topics, such as gambling harms, where stigmatisation is prevalent. Analysis of forum posts can afford in-depth insights that are difficult or impossible to replicate in traditional research settings such as interviews (e.g. [39, 40]). This study therefore utilised online peer support forums to address the gap in knowledge about the stigmatisation of people who experience gambling harms by investigating the experience of stigma in people with lived experience of gambling harm. Specifically, we aimed to answer the following question:

- What are the experiences of stigmatisation and discrimination in a sample of online peer support forum users for gambling harm?

## Methodology

### Data collection and sample

The study was a qualitative study of existing online forum data. Gathering data online presents researchers with an opportunity to collect naturally occurring information on sensitive topics that may otherwise be difficult to obtain [39]. Thus, to gain an insight into the stigmatisation of people who experience gambling harms, data were gathered from three online forums which offer self-help for gambling-related harm. The method of collection and analysis complied with the terms and conditions for the source of the data, and with the BPS guidelines for internet mediated research [41].

The analysis was conducted from a primarily experiential orientation and a critical realist epistemological perspective [42], meaning that the participants’ written language was predominantly used to develop a better understanding of their experiences (experiential orientation; [42]), and that researchers accepted that the data reflected the objective realities of the participants whilst acknowledging that the reality of each participant sits within a cultural, societal, and historical context.

### Forum identification and thread selection

Due to online forums containing tens of thousands of posts, a selection of salient threads was chosen for analysis, which involved two members of the research team searching independently and collating their results to form the final dataset.

A list was created which included all of the UK-based service providers and general discussion boards dedicated to gambling harm support in the UK (through the research team’s knowledge of the gambling service provider landscape, and through Google searches). Next, a member of the research team visually searched each to check whether the threads each forum included were relevant. Forums were excluded if they required a login to access (i.e. if they were considered private spaces), were not primarily based in the UK or had no relevance to the focus of the research (e.g. self-stigmatisation/experienced stigma, perceived stigma/discrimination). Once these forums had been filtered out, three forums were identified to be relevant (two service providers and one general discussion board). Then, a more strategic approach was taken. The criteria for selection of posts were as follows:

- Recent (i.e. posted in the previous 6 months; identified by sorting posts by ‘most recent’)
- Thread has some level of interactional engagement (i.e. has two or more replies in addition to the initial post)
- The original post was by someone with lived experience of gambling harms (rather than ‘affected others’ or spam/advertising type posts)

Sampling was conducted across a range of main topics and sub-forums to increase the likelihood of gathering data from a varied selection of forum users (as different people may frequent the different areas of the forums). Sub-areas included those dedicated to introductions from new members, recovery diary threads, calls for peer advice/support, and debate/discussion threads.

A list of keywords was developed to help to narrow the focus and identify salient parts of the forums for inclusion. Keywords were also used to search recent threads for relevant content for inclusion (e.g. stigm*, discrim*, judg*, shame, embarrass*, stereotyp*). In addition, non-targeted reading by the first author of the most recent threads was also conducted to identify relevant posts of value to the research questions. This process did not use the keywords.

Additionally, threads that related to help-seeking and its associated obstacles, which are common within such forums [39], and where fear of judgement or stigmatisation is commonly referenced, were focussed on.

In deciding on an appropriate volume of data for analysis, the advice of Braun and Clarke [43] was followed, i.e. the number of threads included was based on what has been sufficient for prior studies to generate useful insights [39], and then the appropriateness of the sample throughout coding was monitored to "make an in-situ decision about the final sample size, shaped by the adequacy (richness, complexity) of the data for addressing the research question" ([44] p211).

#### Final selection

Through this process, the final dataset included 27 whole threads, and 389 posts (approximately 49,000 words) in total, sampled from across three forums (two UK-based gambling peer support forums and one general discussion forum with a sub-forum devoted to gambling peer support–organisation names withheld for anonymity). The length of posts ranged from 1 sentence to over 2,000 words, with most consisting of 1–2 paragraphs of text.

### Theoretical approach and data analysis

The data were analysed based on reflexive thematic analysis (see below; [42, 43]), though to ensure the analysis was robust, reliable and free from undue researcher bias, a trustworthiness protocol (based around that of Amankwaa, [45]) was followed in order to maximise the credibility and rigor of the analysis. This protocol enhances the reliability and validity of qualitative research by focusing on four key criteria: credibility, transferability, dependability, and confirmability. To ensure credibility, the researchers immersed themselves in the data through repeated reading of the forum transcripts, utilised multiple forums to capture a diverse range of experiences, and consulted our lived experience panel to discuss the findings and seek their feedback on our interpretations. Transferability was addressed through thick descriptions of the research context and purposive sampling, enabling readers to determine if the findings are applicable to other settings or groups. Dependability was maintained by keeping thorough records of research decisions and activities and checking the consistency of data analysis over time through coding and recoding. Confirmability was achieved through reflexivity, where the researchers acknowledged and addressed their own biases, and through confirmability audits involving members of the research team which were not involved in the data collection or initial analysis reviewing the data. Scrutiny and debriefing sessions were integral to the research processes. Scrutiny sessions involved presenting research methods and preliminary findings to all members of the research team for critical evaluation, helping to identify and mitigate potential biases and enhance the overall credibility of the study. Meanwhile, debriefing sessions facilitated reflective discussions among the research team, allowing for collaborative interpretation of findings, addressing challenges encountered during the research, and ensuring consistency in data analysis. These sessions not only improved the quality and reliability of the research but also promoted a deeper understanding of the data and its implications.

Analysis was primarily inductive, though there was focus on identifying codes and themes which would elucidate factors driving perceptions/stereotypes/stigmatisation.

Thematic Analysis (TA; [43]) was identified as the most appropriate analytic approach as it allows flexible, data-driven analysis, rather than one tied to a specific theoretical framework, which encourages a structured approach to analysis [46]. Thematic analysis allows for a good overview and summary of a large amount of data and has the potential to generate “unexpected insights” which is useful for understudied areas such as the focus here [47]. Thus, the analysis is informed by—rather than being driven by—existing theory.

The forum threads were first imported into NVivo 12 and analysed in accordance with the guidelines as described by Braun and Clarke [44] as follows:

1. Familiarisation of data through repeated reading; at this stage anything interesting or significant was noted, and potential themes recorded in a research diary.
2. Initial coding; the most salient segments of data were inductively coded using ‘essence-capturing’, evocative names, grounded in the language of the users [48]. Iterative re-reading of the threads and subsequent code refinement occurred systematically, allowing the first author to progressively condense, organise, and enhance their interpretation of the data through recursive cycles
3. Searching for patterns; after coding the data, the primary author engaged in a process of reading and reflecting upon the codes and the corresponding data extracts. This deliberate effort aimed to actively interpret recurring, coherent, and meaningful patterns within the dataset, with the goal of identifying core ideas that could unify the observed patterns (i.e. ‘central organising concepts’; ([48] p236). This resulted in seven prototype themes and many related subthemes.
4. Reviewing themes by returning to the original data set and comparing the themes against it; during this phase, extensive discussions took place within the broader research team regarding the identified patterns. These discussions revealed multiple nuanced interpretations of the data, contributing to a more refined understanding. Deliberations on the themes within the research team not only fostered enhanced coherence but also deepened the analysis. Subsequently, the themes were reorganised to better reflect the insights gained from these collaborative discussions and were once again subjected to examination and consideration by the research team.
5. Defining and naming themes; this process was repeated until each researcher felt that the themes sufficiently represented what had been written by participants in the threads, and provided a compelling, thoughtful, and ‘interpretive… story about the data’ ([44] p. 339). The themes were named using concise and ‘catchy’ titles which provided a ‘vivid sense’ of their ‘essence’ ([48] p.258).
6. Producing the report; the results were written up in an integrated and logical order. Extracts of coded data are presented throughout the analysis in order to substantiate the analytical assertions made, and to provide clarity and depth of meaning to the reader. Identifying information has been removed, including forum poster handles which have been replaced with participant numbers (e.g. Ppt 1).

The qualitative analysis was conducted on both semantic and latent levels to comprehensively understand the data. At the semantic level, the focus was on the explicit content of the text, examining the surface meanings and identifying clear patterns, themes, and categories based on the direct responses and observable details. This involved coding the data to capture precise words, phrases, and concepts articulated by the participants. Concurrently, at the latent level, there was a deeper exploration into the underlying meanings, interpretations, and assumptions that might not be immediately evident. This involved interpreting the underlying context, inferring the broader implications, and uncovering the hidden structures and deeper significance of the data. By integrating both levels of analysis, a nuanced and thorough understanding of the qualitative data was achieved, capturing both the explicit and implicit dimensions of the participants’ experiences and perspectives.

The authors contributed a diverse range of perspectives to the study, leveraging their varied backgrounds to enhance the research. Gambling harms research is the dominant area of expertise for KP and JL, and KP has lived experience of gambling harms as an affected other. LN’s research focus spans Education and Cyberpsychology, and DDC’s primary area of expertise is intellectual disabilities. All authors have worked in gambling harms research and all share an interest in understanding the experiences of stigmatised and marginalised communities.

All members of the research team were involved in the analysis of the data. The first and last author conducted initial forum searchers; the first author checked threads for relevance and complied the dataset, and completed initial coding of the full dataset. The second, third and last authors each coded a small sample of data independently before discussing and comparing codes in a consensus meeting. The first author developed the initial themes, which were then discussed and refined by all authors. The first author wrote the first draft of the paper, and the other authors reviewed and edited the final draft.

### Ethical considerations

Ethical approval was obtained from the School of Psychology Ethics Committee at the University of Wolverhampton. As this study did not involve obtaining informed consent from participants, the data were obtained from publicly available threads which did not require a password, registered account, or confirmation of age to access [49]. Permission from administrators was not sought. Data were anonymised by removing identifiable information and usernames [49]. Verbatim text from forums was used for analysis, but in order to protect confidentiality of forum posters, some illustrative quotations presented in the findings here have been minimally paraphrased, where needed to ensure the original posters are not traceable online (success of this process was checked by entering the quotations into search engines and ensuring the results did not include the forum where the original quote originated). An inter-rater consensus process was followed to ensure meaning and tone of paraphrased quotes remained true to the original meaning (practice in line with BPS Internet Mediated Research guidelines; [41]. This involved a second researcher reviewing the paraphrased quotes against the originals, and confirming that they captured the meaning accurately. Where there was disagreement, both researchers consulted to revise the quote until both were satisfied that the paraphrased version sufficiently retained the meaning of the original.

## Results

Through analysis and repeated coding five core themes were identified within the data. There were connections and overlaps between the main themes, and several subthemes were identified within each theme. An overview of the themes is presented below, followed by a detailed analysis of the themes and their related subthemes, illuminated through quotes from the transcripts.

### Overview of themes

Participants’ forum contributions centred around five themes: 1) beliefs about the nature and origin of gambling addiction, relating to participants’ beliefs about causes of gambling harm and cognitions about the nature of ‘addiction’; 2) self-stigma, which encompasses the frequent and substantial incidences of self-stigma–both explicitly communicated and implicitly conveyed, e.g. through language; 3) anticipated stigma, which describes the stigma and discrimination people expected to face from others because of their gambling harm; 4) stigmatising other people who experience gambling harms, which includes the ways in which people who experience gambling harms sometimes stigmatise other people who experience gambling harms; and 5) experienced stigma and discrimination, which encompasses how people experienced overt stigmatisation/discrimination by others in various scenarios.

### Theme 1: Beliefs about the nature and origin of gambling addiction

This theme relates to participants’ beliefs about causes of gambling harm and cognitions about the nature of ‘addiction’. Understanding participants’ beliefs about the nature and origins of gambling harm is crucial because these beliefs contextualise subsequent stigmatising thoughts, emotions, and behaviours towards people with experience of gambling harm. Moreover, the beliefs themselves can be stigmatising. For instance, the belief that gambling harm is rooted in a moral dichotomy suggests a stigmatising perspective that people who experience gambling harms are inherently ’bad’ individuals, which further implies a degree of blame.

Ways of thinking about gambling harms underpinned how people understood and made judgements about their own experiences of gambling and harms, and frequently contributed to self-stigma. This, in turn, influenced people’s expectations about how other people would view them because of the gambling harms they experience (i.e. their ‘anticipated stigma’). It also impacted how individuals perceived (and potentially stigmatised) other people with experience of gambling harm. Thus, the beliefs are presented here to highlight stigmatising attitudes about the nature of gambling harm, and to provide context for the various types stigma identified in the analysis (and which are discussed further in themes 2, 3, 4 and 5).

The over-arching theme comprises four subthemes: pathologisation, parallels with substance use, moral binary, and the embodiment of gambling as the enemy.

#### Pathologisation

There was frequent pathologisation of gambling harm, with references to gambling harm as an *“illness”* or *“disease”*, an *“obsession/compulsive behaviour”*, or an *“unhealthy habit”*. There was variation in beliefs about the nature and extent of gambling harm as an illness. Some people described it as a “*progressive*” illness which “*will only get worse until you stop*”. Others held the belief that one can experience improvement by exchanging “*unhealthy habits*” for healthier ones. There was variation in how enduring people believed gambling harm to be, based on which one of these labels they assigned to it. For example, the people who believed gambling harm to be an illness or disease typically believed it to be chronic and lifelong:

*“My wife came to terms with what I had done with some counselling from [gambling support provider] and thinking of my addiction as an illness which I will always be recovering from.”* (Ppt 34)

This quotation not only demonstrates that Ppt 34 and their wife consider gambling harm to be an illness, but that they were encouraged to think this way by a gambling support provider. In this instance it also allowed the person’s wife to begin to accept the situation. This implies that if gambling is framed as an illness rather than a choice, that there is absolution of blame for the individual, which can be beneficial. However, considering it as something which one will “*always be recovering from*” could also diminish hope, as some people who described it in this way consequently felt as though they had no chance of getting better or ‘recovering’ completely.

Predominantly, people used addiction-focussed language *(“addiction”/ “addict”)* when speaking about themselves and others experiencing gambling harms. The use of this language is complex and seems to serve different functions in different contexts. People described addiction as both something one has (in the same way someone would describe having a physical illness i.e. ‘I have asthma’), something you are (i.e. *“I am an addict”*), and as a separate entity (i.e. *“the addiction is pretty strong”*). As with the disease/illness framing, when people spoke of gambling addiction as something they have, there was an implicit suggestion that it was not their fault; they perceived it as an affliction or a sickness. Characterising addiction as a distinct entity also sometimes served to absolve individuals from blame, as expressions such as "*the addiction got the better of me*" conveyed a sense of being outsmarted or overwhelmed by an external force that was seemingly intent on disrupting their lives, despite their reluctance and desire to resist its influence. However, people frequently used the label “*addict*” to describe both themselves (“*an addict like me”)* and to label other people who experience gambling harms *(“we are gambling addicts”*) which had a different effect. By labelling themselves and others as *“addicts”*, there is an implicit suggestion that there is an inherent flaw or issue within the individual, for example:

“*Welcome to hell. You have a girlfriend and 12k. At least for now. That’s more than most gambling addicts will ever have*.” (Ppt 111)

In this quotation, Participant 111 not only uses the term ’addict’ to characterise another forum user, but there is an implication that due to this label, the individual’s circumstances will never improve; it suggests a belief that those identified as ’addicts’ are destined to persist in their current state, unable to secure financial stability and maintain relationships. This use of addiction-first language also reduces the person to their gambling harm and, more broadly, implies that all ‘addicts’ are the same, placing them into a stereotyped group. As such, parallels with substance use can be drawn, which will be discussed below.

#### Parallels with substance use

Participants drew parallels between gambling and Class A drug use (particularly ‘crack cocaine’ but also heroin). Parallels were drawn both between gambling and using drugs, and people who experience gambling harms and people who use drugs. Sometimes, this was done to emphasise the seriousness of difficulties associated with gambling harm:

*“Slots are my crack cocaine … I am mathematically challenged but after being given that hit of “crack” I have been desensitized” (Ppt 174).*

In an effort to underscore the gravity of the gambling impulses experienced by Participant 174, an analogy is drawn between slot machines and ’crack cocaine,’ elucidating the development of a tolerance to the effects of gambling in a manner analogous to the tolerance observed in individuals who use drugs. Other times though, parallels were drawn in a derogatory way, and the parallels drawn between people who experience gambling harms, and drug users served to invoke a merging of stigma from one stigmatised identity to another. There were various references to feeling, being or behaving in an such a way which was deemed similar to assumed behaviour of people who use ‘crack cocaine’. For example:

*“I was behaving like a crackhead to be honest” (Ppt 86).*

Beyond simply drawing parallels with another form of addiction, the choice of the term ‘crackhead’ holds significance, given the heavy racialization and criminalization seen in discourses around crack use (e.g. [50]). Whilst the word ‘crackhead’ is in itself dehumanising and stigmatising (of the self and those being stereotyped), this quotation also implies that individuals who use drugs exhibit reprehensible behaviour; which is out of character for the poster, but who has been driven to it by gambling. Further, use of the word ‘crackhead’ was often preceded with the word ‘dirty’ (*“I just feel like a dirty crack head”*, Ppt 107*)*, implying an inherent negativity associated with people who use ‘crack cocaine’, insinuating and that they are somehow tarnished and unclean. This simplistic dichotomous perspective relates to the next subtheme, moral binary.

#### Moral binary

Gambling as an activity, and by extension, people who experience gambling harms, were spoken about in terms of a moral binary, primarily when people where speaking about themselves rather than others. People frequently described gambling in terms of right or wrong and good or bad:

*“For a while I was good having no money and was decided to quit. But now, I chose the wrong decision again”* (Ppt 144)

“*I was good for 4 months*” (Ppt 172).

The first extract exemplifies how people believed gambling to be ‘wrong’, and both together illuminate how this belief, when coupled with continuation of gambling, affects sense of self. The use of the phrase "*I was good for quite some days*" implies that, during periods of gambling, individuals perceive themselves as ’bad’. The forums were also littered with references to being or becoming “*clean”* or “*dirty*”. The dualistic view that people can be classified as ‘clean’ or ‘dirty’ is a common conceptualisation in many aspects of life and is grounded in an idealised cognitive model where dirt symbolises disorder, while cleanliness represents order [51]. Thus, by drawing similarities between people who experience gambling harms and ‘dirty crackheads’, there is an implication that people who experience gambling harms cause/embody disorder.

Religious terminology was used frequently, encompassing allusions to ‘demons’, ‘the Devil’, ‘Hell’, and a sense of forsakenness by ‘God’ (expressions which could be regarded as the epitome of moral dualism). Some people felt as though their gambling was being guided by malevolent force which they had no control over. For example:

*“I’m trying to change my behaviour, getting the split personality feeling of not knowing who I am, there’s a devil on my shoulder.” (Ppt 174)*

The above quotation describes a perception of a self-division, wherein one part embodies the individual, and the other is purportedly influenced or controlled by a metaphorical ‘Devil’. This could be construed as emblematic of a conflict between ‘good’ and ‘evil’. This sense of conflict resonates throughout the forums, though the most striking references to religious ideology are feelings of abandonment by ‘God’ or attributing gambling harms to what is perceived as an expression of ‘God’s wrath’.

*“Gods not watching over no one’s degenerate bets that’s for sure. Devils always lurking tryna to get you to bet it all. God watching over someone betting that’s funny stuff” (Ppt 138)*

*“it’s a sign from God that what the future holds for me in betting is suffering.” (Ppt 52)*

*“May God have mercy on me*. *Please don’t let me gamble again*. *I must have been an evil person [in a] past life to have this life*. *I hate myself” (Ppt 47)*

These quotations illustrate how people perceived the ‘abandonment by God’ as evidence that they are ‘degenerate’ and ‘evil’ inside or that they are deserving of punishment because of some moral failing. The first quotation includes a wry comment about how “*God’s isn’t watching over anybody’s degenerate bets that’s for sure*” which implies that all people who gamble have been cast out by God, and is explicitly stigmatising. It also connotes a resigned despair suggesting that people who experience gambling harms are powerless to ‘the Devil’s’ temptation. Overall, these quotations also highlight a sense of self-loathing and low self-esteem as the result of classifying gambling harm in this dualistic way. The feeling of being powerless over gambling feeds into the next subtheme, the embodiment of gambling as the enemy.

#### The embodiment of gambling as the enemy

The portrayal of gambling was articulated in a way that anthropomorphised it, characterising it as some monster or ominous being by using descriptive words such as “*beast*” or “*demon*”. Further, this *“beast”* was perceived as so strong and powerful that the only options were to ‘run’ (*“run from it [gambling]*, *good person—run fast and far away from it”*, Ppt 109*)*, or try to ‘fight’ (“*strike now*, *quickly*, *while you are feeling strong”*, Ppt 10). This final quotation suggests that individuals may only possess the capacity to overcome the adversary momentarily, exhibiting a fleeting strength that is destined to diminish over time. Experiencing gambling harms was also more directly compared to a physical fight:

*“A person said here that in his ga* [Gamblers Anonymous] *room they say if you had a fight in the ring with Mike Tyson and obviously got beaton* [sic] *to a pulp very quickly and lost and was very badly bruised after. Would you ever get back in the ring again? For many of us gambling is Mike Tyson. It destroys us every time. Why on earth get back in the ring? Let’s all hang up our boxing gloves.”* (Ppt 16)

In this analogy gambling is again constructed as vastly stronger and more powerful, and that any attempt to control it is futile. Overall, there was a sense of despair and helplessness which was present throughout the forums, as though the battle were hopeless with no option but to admit defeat.

### Theme 2: Self-stigma

This theme encompasses the frequent and substantial incidences of self-stigma, and there are close links with several other themes. Often self-stigma is related to stigmatisation of other people who experience gambling harms, where a negative perception of oneself is generalised to others perceived as falling within the same ‘category’, and vice-versa (*“we don’t have the mentality to stop”*, Ppt7*; “gamblers lie*, *cheat and deceive so don’t believe anything”*, Ppt 10). Sometimes self-stigma fed into anticipated stigma; people predicted, based on their own feelings about themselves and the gambling harms they have experienced, that others would react negatively to them if/when they disclosed their experiences. In one example, someone’s self-stigma made them feel unworthy of a non-stigmatising response from a loved one (*“I told my mom and dad today*! *I’d played it over and over in my mind… [but] mom was more reasonable than I’ll ever deserve”*, Ppt 23).

The over-arching theme is grouped around three domains: negative cognitions about the self, negative self-talk, and negative emotions towards the self.

#### Negative cognitions about the self

The beliefs people had about the origins and nature of gambling harm find reflection in the way people perceived themselves and their gambling, describing themselves in ways such as “*broken*”, *“evil”*, *“unclean”*, *“bad”* and *“fucked up”*. For example, Ppt 5 said:

*“Was I evil, dumb, stupid or just no good”* (Ppt5)

This quotation encapsulates the negative perceptions people had about themselves, illustrating how when some searched for reasons for their gambling harm, they concluded that a fundamental flaw within them was responsible. Beliefs about parallels with substance addiction and those who experience harms from substance use were also apparent:

*“I’ve told my family numerous times in the past, I just feel like a dirty crackhead and at face value to people I’m seen as an ‘intelligent’ young man yet I’m clearly the opposite, I’ve been lying to my girlfriend for years but it’s coming to the point I can’t hide it.” (*Ppt 107*).*

Linked to the theme 1, parallels drawn between people who experience gambling harms and people who use drugs serves to invoke a merging of stigma from one stigmatised identity to another. Further, the above quotation implies that people who experience gambling harms are unintelligent. Finally, and similarly linked to theme 1, people often believed they were deserving of punishment, because of the negative labels they had associated with themselves (*“I am a loser and don’t deserve anything”*, Ppt 52).

#### Negative self-talk

People spoke to and about themselves with a substantial–and sometimes quite extreme—degree of criticism and self-deprecation. which included people describing themselves as *“arrogant”*, *“broken”*, *“childish”*, *“cowardly”*, *“degenerate”*, *“evil”*, *“fucked up”*, *“greedy”*, *“loser”*, *“mean”*, *“pathetic”*, *“selfish”*, *“stubborn”*, *“stupid”*, *“trash”*, *“useless”*, *“weak”*, *“whiny”* and *“worthless”*. For example, Ppt 174 described frustrations at his continued gambling despite the negative repercussions, resulting in an extremely low opinion of the self, ascribing very negative labels to themselves:

*“I don’t like gambling I am fine without it. I don’t understand why I do this to myself it makes me feel pathetic and worthless just a degenerate gambler with nothing left to lose.” (Ppt 174)*

The words *“I don’t know why I do this to myself”* insinuate a high level of self-blame attributed to being *“just a degenerate gambler”*. This quotation further illustrates the internal conflict highlighted in theme 1, wherein the individual experiences a sense of compulsion to gamble despite a genuine desire to stop. Another described themselves as “*the biggest trash on earth*” (Ppt 47), illuminating the extremely low self-concept they hold.

#### Negative emotions towards self

The negative cognitions and self-talk resulted in people experiencing strongly negative emotions about themselves, including shame, guilt, embarrassment, disgust, loathing, and hatred, as illustrated in the following quotations:

*“I hide any cups or gaming tickets that might divulge where I was I know I hate the feelings…the self-loathing. Maybe I barely slept and am worthless the next day.” (Ppt 181)*

*“It makes me feel pathetic and worthless” (Ppt 174)*

*“I’m disgusted at myself” (Ppt 47)*

*“I’m over 50 and I haven’t got 2 pence to rub together it is pathetic*. *I feel completely pathetic*.*” (Ppt 54)*

These quotations encapsulate the sometimes-extreme negative feelings people felt towards themselves, and feelings of incredibly low self-worth expressed through–and potentially created by—harsh self-criticism. They also demonstrate again the internal conflict people face as their actions do not meet their expectations of themselves. Shame was a particularly prevalent self-emotion. People felt ashamed of their gambling and its consequences, ashamed about concealing problems, ashamed about lying and ashamed about the impact their gambling would have on their families. For example, one participant expressed an incidence where they felt that (when talking about an incident where they had relapsed), would be a disappointment to their family, saying:

*“I feel so ashamed, horrible and just feel like I’ve let my family down again”* (Ppt 39).

These feelings of shame and worry about the reactions and impact of gambling on individuals’ families relate to theme 3 (anticipated stigma), which will be discussed in more depth below.

### Theme 3: Anticipated stigma

This theme describes the stigma and discrimination people expected to face from others because of their gambling harm. Often linked with theme 1 (beliefs about the origin and nature of gambling harm) and theme 2 (self-stigma); people expected others to share their thoughts and feelings about people experiencing gambling harms, i.e. their expectations about what kinds of thoughts and feelings others would have about them was dictated by their own beliefs about gambling harm–particularly beliefs about causes and blame, and perceptions of gambling addiction as a permanent and enduring disorder and/or character flaw. This theme had three subthemes, which were fear of disclosure, expectations of how they should be treated and the impact of anticipated stigma.

#### Fear of disclosure

Some were so apprehensive about the potential stigma they might encounter that they refrained from sharing their problems with anyone. There was a dread that people may inadvertently discover their problems, coupled with a reluctance to explicitly tell people. The most prominent concern was that disclosing their gambling to family members would result in rejection (*“If I tell the truth*, *people will leave me”*, Ppt 142). Additionally, there were concerns about the repercussions of their gambling on their families (“*I feel terrified about how this is going to affect my partner*”, Ppt 31), and feeling as though they’d let them down:

“*Nobody has any clue about my addiction… I’ve gambled in secret for years. The shame and the pain of constantly letting my family down behind their back is taking such a toll.”* (Ppt 43)

*“I feels so ashamed about this and I need to tell him but I think that this is going to devastate him*. *I believe that this will likely end our relationship”* (Ppt 31)

The above quotations highlight the profound sense of shame associated with gambling people in the forums felt, rendering people hesitant to disclose their problems, despite an underlying desire to do so. They also suggest that concealing gambling issues carries a significant burden, leading to considerable distress and as having a substantial impact on wellbeing (*e*.*g*. *“Telling so many lies and living in so many fears is very stressful to our wellbeing”*, Ppt 5).

#### Expectations of how they should be treated

People often thought they were deserving of the stigma they anticipated, believing they deserved punishment (“*I deserve this Hell”*, Ppt 69*)* and were undeserving of having families (*“I don’t deserve my wife and kids”*, Ppt 47*)*, or indeed anything at all (“*I am a loser and I don’t deserve anything*”, Ppt 52). Thus, the anticipation of stigma was intensified, and the apprehension of revealing their challenges became more profound. They perceived that by withholding their problems, they were prolonging an inevitable and justified retribution. When gambling *was* disclosed, there was a belief that they were unworthy of any kindness or support extended to them, for example:

*“My mum, although disappointed and devastated at first, cuddled me, allowed me to just cry…then was more reasonable than I will ever deserve.” (Ppt 173)*

#### Impact of anticipated stigma

For most, the fear of disclosure and expectations people had about how they should be treated meant they kept their gambling harms secret, preventing them from seeking help, despite experiencing significant harms. Gamblers Anonymous was the only formal form of help which was discussed, and, despite some expressing how helpful the meetings had been for them, there was a deep sense of trepidation about attending (“I’ve got my first meeting tonight and I’m s*#tting myself”, Ppt 36). The few that did attend expressed a sense of surprise and relief to feel accepted by other members; a way they had not felt previously (“*No judgement*, *probably for the first time in your life”*, *Ppt 24)*.

For many individuals however, the forums served as the initial or sole platform where they disclosed their problems. Because of this, there was a sense of desperation and panic as people grew increasingly concerned that they would no longer be able to conceal their gambling (*“I’ve been lying to my girlfriend for years but it’s coming to the point where I can’t hide it”*, Ppt 107). The fear and reluctance to disclose problems to family or seek help posed a significant emotional burden, and suicidality was frequently discussed as people felt they had no other choice:

*“My parents/friends don’t know my problem and I have considered suicide 3 or 4 times this year*” (Ppt 62)

*“I’m left with this idea that suicide is probably the only option”* (Ppt 113)

Thus, the potential consequences of anticipated stigma were sometimes so severe they posed a direct threat to life.

### Theme 4: Stigmatising other people who experience gambling harms

This theme describes the ways in which (some) posters who experienced gambling harms stigmatised other people who experienced gambling harms. Unlike the incidences of self-stigma described in theme 2, there were very few incidences of overtly stigmatising language being used towards other people who experienced gambling harms, though the same addiction-focussed and gambling-first language as described in theme 1 was used to label others (“*we are gambling addicts*”, Ppt 55). This was in stark contrast to the extent of negative self-talk. There were however frequent examples of people explicitly trying to challenge others’ self-stigma. This sometimes led to scenarios where people stigmatised themselves but lifted others up (e.g. *“Don’t judge yourself… stand tall… you have done something I never could have done [self exclusion]”*). Where incidences did occur, the cognitions people who experience gambling harms had about other people who experience gambling harms often reflected negative perceptions of the self which were generalised to the group as a whole:

*“It’s hard for non-gamblers not to pass judgement about the things that we, as gamblers, have done”* (Ppt 60).

*“Like every gambler*, *I’ve been deceitful”* (Ppt 70).

This may have been a deliberate attempt to avoid overtly stigmatising people within the forum, given the social context and development of rapport between forum posters. In addition, stigmatisation of others, when it occurred, echoed self-stigma in that it involved stigmatisation though cognitions, talk and emotions about others.

#### Cognitions and emotions about others

Some believed other people who experience gambling harms to be emotionally volatile, that they “*lie*, *cheat and deceive*”; and that they “*whinge*” but “*don’t really want to do anything about it*”. There was also a belief, by some, that people who experience gambling harms lack intelligence, and that they are untrustworthy which, in one instance, was given as a reason for not going to Gamblers Anonymous meetings:

“I never went to any “meetings”—those f***ers were trying to win my money; I was trying to win theirs” (Ppt 154)

People also made stereotypical assumptions about other people who experience gambling harms and the characteristics they possessed. For example, when talking about the expectations of attending their first Gamblers Anonymous meeting, one person said *“I assumed it would be full of scum”* (Ppt 23).

People spoke very infrequently about their emotions towards others experiencing harms; there was a sense that most of the posters were experiencing a ‘crisis point’ where they were experiencing severe distress but hadn’t told anyone (linked to theme 3). Because of this, most people were searching for help and were focussed on themselves. One way posters did express negative emotions towards other people experiencing harms was through descriptions of encounters or experiences in their lives, and their reactions:

*“Advice from persistent losers on what to bet on. They’d even spit on the* [bookies’] *floor. How depressing.” (Ppt 37)*

### Theme 5: Experienced stigma and discrimination

The last theme encompasses the experienced stigmatisation/discrimination that some forum users described encountering. This involved being the recipient of negative treatment (sometimes consisting of actions, sometimes of verbal interactions) that arose because of the person’s status as someone experiencing gambling harms. For many posters, there was little to no discussion of experienced stigma or discrimination, because they had not yet disclosed their gambling harms to anyone outside of the forum. For these, predictions about how those around them would react contributed to the theme of ‘anticipated stigma’, instead. When it occurred, experienced stigma or discrimination was often perpetrated (intentionally or inadvertently) by someone without experience of harms, including friends, family, members of the wider community, and in one example, by a gambling industry employee. This theme had three subthemes which were non-hostile discrimination, hostile discrimination, and familial rejection.

#### Non-hostile discrimination

People were often discriminated against in a non-hostile—and not necessarily overtly negative–way. For example, having their partner/parents take control of finances due to the consequences of gambling harm, both as a way to support them, and because of issues around trust. Some posters were content with their partners taking control of things, as they felt it would help them to rebuilt trust and improve relationships:

*“I’ve always said she can look through my bank statements, my loans, my dairy, anything she wants at any time as I want her to trust me”* (Ppt 32)

However, whilst this was sometimes experienced as a practical solution, it could also infantilise people, removing their autonomy, for example, by having to relinquish control of their finances:

*“I can’t access money in any of our accounts. After a while, she told me I could have my credit card back for spending, but she monitors it closely”* (Ppt 34)

While we have termed this type of discrimination ‘non-hostile’ to contrast it with overtly confrontational discrimination described in the next sub-theme and acknowledge that it was sometimes reported as supportive by posters, it does encompass treatment that could be described as problematic–namely, coercive control.

#### Hostile discrimination

Whilst there were few incidences of people reporting experiencing hostile discrimination because of gambling harms, those that were reported were powerful and poignant. One described going to a casino to self-exclude and being “*humiliated*” by the manager, who made them justify their request in front of other customers:

*“So tonight I decided to finally have the courage to go to my local bingo hall and self-exclude myself. Well I wish I hadn’t of bothered. To say I was humiliated is an understatement. I was told to sign in which I didn’t want to do, I was then taken to one side in-front of lots of people who could clearly hear the manager speaking to me about my issues and asking questions about why I wanted to self-exclude. This is exactly the reason why I have put it off for so long. I’m so upset I ended up walking out not even doing it. I went home and cried and actually feel worse for trying to do it. I felt I had turned a corner as I haven’t gambled in a few weeks and now I just feel like utter rubbish.”* (Ppr 19)

In this instance, the woman not only felt humiliated, but the manager’s behaviour also significantly affected her emotional state. Her statement, "I felt like I had turned a corner" by not gambling, contrasted with her current feeling of "utter rubbish," implies that this interaction has disrupted her recovery progress and negatively impacted her emotional well-being.

Another person described being repeatedly *“disgraced”* over a loan, overlooked for work (“*No one will give me work anymore”*, Ppt 52), and being physically assaulted by someone while immersed in a session of online gambling (*“I was beaten…* [by] *a man who kept hitting and slapping me”*, Ppt 52).

This last example starkly illustrates that, in their most extreme forms, stigmatisation and discrimination related to gambling harm can escalate to violence. Such instances underscore the profound and sometimes brutal impact of societal attitudes toward individuals affected by gambling harm. These were the only reported incidences of overt hostile discrimination from the forum data.

Whilst this subtheme contains few examples of hostile discrimination, it is included due to the significant and critical insights it provides. Despite its infrequency, the severity and consequences of hostile discrimination warrant attention and emphasis.

#### Familial rejection

The fear of rejection described in theme 3 was realised for some, who described feeling that their families were ashamed of them, or that they ‘hated’ them, and ultimately some were rejected by their families after disclosing gambling harms:

*“Today…. I told my parents! It was honestly one of the most horrific experiences of my life. I’d played the scenario over and over in my head for months. There was lots of shouting, and nasty comments. Even more tears. My dad would barely look at me and didn’t say “I love you” back when I left.”* (Ppt 23)

However, in another post, the person did describe a sense of relief at having disclosed their problems:

*“Tonight was the first time I’ve actually spoken aloud about the mess I’ve caused. I feel like I can breathe for the first time in forever.”* (Ppt 23)

In this case, disclosure to family was the catalyst for the person seeking more structured support (“*I am attending my first GA meeting tomorrow”* Ppt 23). However, very few posts described people actually making the step to disclose problems outside of the forums; most posts described an intense apprehension about having to tell family based around the expectations of how they would react (theme 3), and that they were unable or unwilling to do so.

### Timeline

Experiences discussed within the forums loosely fitted around a timeline of gambling harms. This was developed in the research team’s scrutiny and debriefing sessions into a tentative timeline which was reviewed by the lived experience panel and deemed a useful chronological representation of gambling harms and their associated stigma. This inference was drawn from the topics discussed by individuals at different stages of recovery, rather than explicit references to a timeline or acknowledgment of phase-specific experiences. It is more implicit than explicit, making it challenging to illustrate directly with quotes.

**Fig 1:**
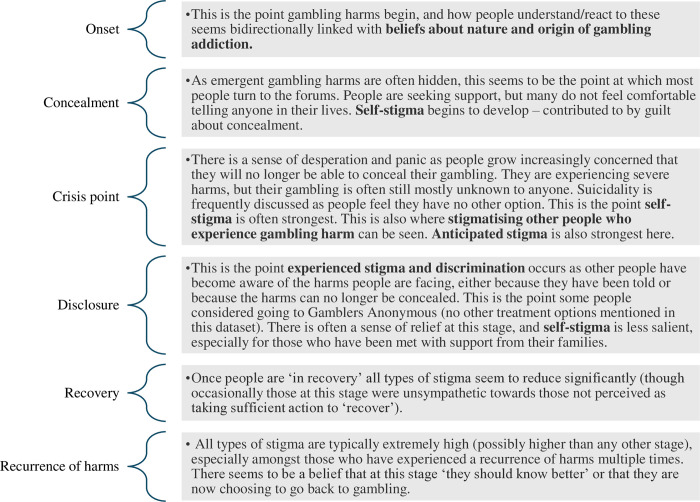
Tentative timeline of stigmatisation.

The timeline involved six stages: onset, concealment of problems, crisis point, disclosure of problems, recurrence of harms (sometimes termed ‘relapse’) and recovery. (The themes and subthemes from the analysis map onto the timeline, and have been emboldened in Fig 1 to illustrate where they fit). Those posting on the forums were at different stages, and not all had experienced all six stages–e.g. some had never disclosed gambling harms outside the forum, and some had not experienced recurrence of harms. Some had cycled through stages more than once, from recurrence of harms back to concealment, crisis, or disclosure and again onto recovery. Hence though conceptualised here as a timeline, these stages and their potential to be iterative could also be conceptualised as a cycle or iterative process of gambling harm. Experiences of stigma both impacted, and were impacted by, progression along the timeline: E.g. prolonged concealment was frequently driven by anticipated stigma, and recurrence of harms was a trigger for heightened self-stigma and experienced stigma.

## Discussion

This study explored the experience of stigma in people with lived experience of gambling harm through the thematic analysis of naturalistic data from online peer support forums. The analysis described five main themes relating to beliefs about the nature and origins of gambling addiction, self-stigma, anticipated stigma, stigmatising other people who experience gambling harm and experiences of stigma and discrimination.

There were varying views on the nature and origin of gambling addiction but primarily people described it as an illness or disease, and felt it was akin to drug dependence. In line with research that has evidenced considerable stigmatisation of people who use drugs (e.g. [52]), posters who compared themselves with people who experience harms from drug use typically did this in a self-deprecating, stigmatising manner. Thus, while there is a lack of consensus as to whether the ‘disease’ model of addiction increases or decreases stigma, and various factors including culture are likely to impact this (e.g. [53]), those in our study who implicitly or explicitly subscribed to this model tended to demonstrate experiencing self-stigma.

There was also an emphasis on a moral aspect of gambling problems which suggested that people had an individualistic perception of who or what was to ‘blame’ for the harms felt; either people experienced harms because they were inherently ‘bad’, or that gambling itself was ‘bad’ or ‘the enemy’. Amongst the former, stigmatisation of themselves and/or others experiencing gambling harm was typically particularly evident, consistent with the wider literature on drug and alcohol use demonstrates that moral ‘models of addiction’ are associated with increased stigma [53]. Individuals’ perceptions of gambling harms significantly shape their understanding of their own experiences, often resulting in self-stigmatisation. This self-stigma impacts their expectations of how others will view them in light of their gambling-related struggles, leading to anticipated stigma. Furthermore, these perceptions influence how individuals perceive and potentially stigmatise others facing gambling harm. These observations align with existing literature on the mechanisms underlying the stigmatisation of gambling harms, such as beliefs regarding the recoverability of gambling addiction and its potential for causing harm or disruption (e.g. [13]). Such beliefs can profoundly influence individuals’ attitudes towards their own experiences, as well as those of others, thereby affecting self-stigma, stigmatisation of others, and anticipated stigma. These findings are also consistent with broader literature on the intricate relationship between beliefs about the origin or cause of a condition, notions of responsibility or ‘blame’, and the processes of stigmatisation [20].

Self-stigma was notably widespread, which is unsurprising given the societal stigma surrounding gambling harms [35], alongside the high co-occurence between gambling harms and depression [54]–both of which could contribute to an individual’s perceiving themselves negatively. While the attribution of negative stereotypes to all gamblers was common, overt stigmatisation of other forum users facing gambling harms was comparatively rare. This could potentially be attributed to social factors such as the tendency to refrain from openly stigmatising others to spare their feelings. Alternatively, it may represent a genuine phenomenon wherein individuals tend to judge themselves and the abstract concept of "gamblers" more harshly than they do real individuals experiencing gambling harms, whom they may have heard about and empathised with.

Experienced members of the forums tended to exhibit lower levels of self-stigmatisation and often encouraged others to be more compassionate towards themselves. Although this study did not follow participants longitudinally, and the assessment of the forum’s role or effectiveness was not a focus, it suggests that self-stigma may diminish over time, especially with engagement in peer support. This observation aligns with the principles of "contact theory," which proposes that exposure to individuals with stigmatised characteristics can effectively reduce stigma [55]. While most research on this theory concentrates on decreasing public stigma among individuals who lack personal experience with the stigmatised characteristic, there is promising evidence indicating that social contact interventions can also mitigate self-stigma [56]. Further investigation into whether such interventions could effectively reduce self-stigma related to gambling harm, particularly through virtual interactions like forums, would be valuable.

Anticipated stigma significantly influenced behaviour, often acting as a deterrent to disclosing or seeking help, as individuals feared encountering the stigmatising reactions they anticipated. It also appeared to exacerbate distress for individuals who had not yet disclosed their experiences of gambling harms to loved ones or professionals, as they lived in fear of potential future stigma. While some individuals described anticipating greater stigma than they actually faced upon disclosure, for others, anticipated stigma accurately foreshadowed the stigma they ultimately encountered. This aligns with broader literature indicating that individuals often conceal their gambling problems due to feelings of shame and fear of judgment [13, 21, 23, 25–27]

There were minimal instances of individuals in the forums stigmatising other people who experience gambling harms, although attitudes and beliefs toward other individuals facing gambling harm were consistent with the self-stigmatising perspectives individuals held about themselves. Similarly, there were few reports of people directly discriminating against them, aligning with existing literature (e.g. [35]), likely due in part to many forum users not disclosing their experiences of gambling harm outside of the forum. Some accounts of being treated differently due to gambling harm were seemingly well-intentioned, such as family members taking control of finances as a practical strategy to control harm. However, reactions to this common occurrence varied, with some individuals feeling infantilised or mistrusted, while others felt supported. While we did not encounter any explicit references to intimate partner violence in the posts we analysed, previous research has identified complex relationships between gambling, coercive control, and intimate partner violence (e.g. [55]). The autonomy of the individual experiencing harm in implementing such strategies for monitoring spending can influence whether this intervention is perceived as discriminatory and controlling, or supportive. Occasionally, individuals reported experiencing deliberate hostile discrimination as a result of experiencing or disclosing gambling harm. In such cases, the experience was distressing and was associated with a worsening wellbeing and self-stigma.

The fluctuations in levels of stigma reported at different time points, particularly the identification of periods of recurrence of harms (’relapse’) as triggers for increased stigma, mirror findings from the literature on individuals experiencing harms related to substance use (e.g. [56]). These findings offer valuable insights for stigma-reduction interventions and professionals in support or treatment roles, specifically: (a) general stigma-reduction campaigns aimed at reducing societal stigma, anticipated stigma, and self-stigma can play a crucial role in encouraging individuals before disclosure to seek support; (b) online peer support groups serve as valuable spaces where individuals struggling with self-stigma and fear of disclosure can begin to receive support while maintaining anonymity; (c) stigma transitions from being ’anticipated’ to ’experienced’ during disclosure, and during this challenging period, reactions to disclosure, particularly from family members, vary and can either exacerbate or alleviate both self-stigma and experienced stigma; and (d) the recurrence of harms (’relapse’) can elicit strong negative reactions from both the individual experiencing harms and those close to them, presenting a time of heightened risk for both self-stigma and experienced stigma. Managing expectations and interpretations surrounding the recurrence of harms may be crucial to enable individuals to cope with it if it occurs, fostering less distress and a less binary (success/failure) perspective.

This study was UK-based as the work was funded by a UK charity, and the scope was to understand experiences in Great Britain. However, it is possible that some forum users were from other parts of the world. Contextual clues from the forums, such as references to sporting events and specific language used, suggested that most participants were from the UK. Additionally, posts clearly from non-UK participants were actively excluded, such as those referencing dollars instead of pounds. As such, the results of this study may not represent the experiences of individuals in countries outside of the UK. The stigma surrounding gambling and gambling harm likely varies between countries due to factors such as differing gambling regulations, religious practices, and social systems. Research in stigmatisation of addiction more broadly has identified, for instance, that different beliefs about models of addition in different countries can influence stigma [53]. Future researchers could replicate this study with an expanded scope to include experiences from individuals outside the UK. Additionally, quantitative studies could explore potential differences in gambling-related stigma across various countries.

We also recognise the importance of exploring the influence of demographics (including ethnicity/religion/culture/gender/age etc.) on experiences of stigma but the nature of the forum data (i.e. only being able to infer this information from occasional clues in the posts) made this difficult. This work was however part of a wider programme of research which has explored this through survey and interviews (publications forthcoming).

### Limitations

This work has several limitations inherent in studies employing this methodology. Firstly, the analysis utilised data from a peer support forum; thus, the experiences of individuals captured in this sample may not necessarily be representative of those of other individuals experiencing harms. Additionally, while forum data provide insights into naturalistic conversations within the ’real world,’ unaffected by researcher expectations, individuals’ interactions are still likely influenced by the forum’s audience, potentially not fully reflecting their true feelings, especially when discussing other individuals experiencing harms. Future researchers might consider conducting one-to-one interviews with people experiencing gambling harm to further deepen understanding in this area. Further, when selecting the data, there may be issues around subjectivity based on personal own biases or interests, potentially leading to a skewed representation of the forum content.

### Conclusions

This is, to our knowledge, the first study to use naturally occurring data to systematically investigate the stigmatisation of people who experience gambling harms. The findings underscored the significant impact of societal stigma on individuals’ perceptions of themselves and others, as well as the challenges posed by relapse periods, which can exacerbate stigma and distress. Importantly, the study highlighted potential avenues for stigma reduction, including information programmes (independent of the gambling industry) targeting societal, anticipated, and self-stigma (which should complement independent campaigns and other policies to prevent and reduce gambling and gambling harms) as well as the valuable role of online peer support groups in providing anonymous support to those struggling with self-stigma and fear of disclosure. Moreover, it emphasised the importance of managing expectations surrounding relapse to foster resilience and a nuanced perspective among individuals experiencing gambling harms. These insights offer valuable guidance for stigma-reduction interventions and professionals working in support or treatment roles, pointing towards the need for comprehensive strategies to address stigma across multiple levels and contexts. Our findings contribute to a growing body of evidence to suggest that people who experience gambling harms experience high levels of stigmatisation, which is detrimental to health and a significant barrier to help-seeking in this population.
